# Modeling genetic mosaicism of the mammalian target of rapamycin pathway in the cerebral cortex

**DOI:** 10.3389/fmamm.2023.1231778

**Published:** 2023-08-09

**Authors:** David M. Feliciano

**Affiliations:** Department of Biological Sciences, Clemson University, Clemson, SC, United States

**Keywords:** somatic mosaicism, focal cortical dysplasia, tuberous sclerosis complex, mTORC1, malformations of cortical development

## Abstract

The capacity to integrate complex sensory cues and to coordinate an adequate behavioral response often requires integration of information within the outermost part of the mammalian brain called the cerebral cortex. The laminar and columnar cytoarchitecture of the cerebral cortex contains neurons that establish proximal and distal connections. Genetically encoded transcription factors ensure the generation of the appropriate number, types, locations, and connections of cortical neurons. However, somatic mutations that alter cortical development provide evidence that post-transcriptional regulation is equally important. An example is that somatic mutations in regulators and substrates of mammalian target of rapamycin (mTOR) are associated with neuropsychiatric and neurological manifestations. mTOR is a protein kinase that phosphorylates substrates that control mRNA translation and anabolic processes. Numerous challenges remain in uncovering the mechanisms by which mutations in regulators and substrates of mTOR impact behavior. Here, evidence is provided that somatic mosaicism can be modeled in the developing murine cerebral cortex which may have clinical significance.

## Introduction

**FIGURE 1:**
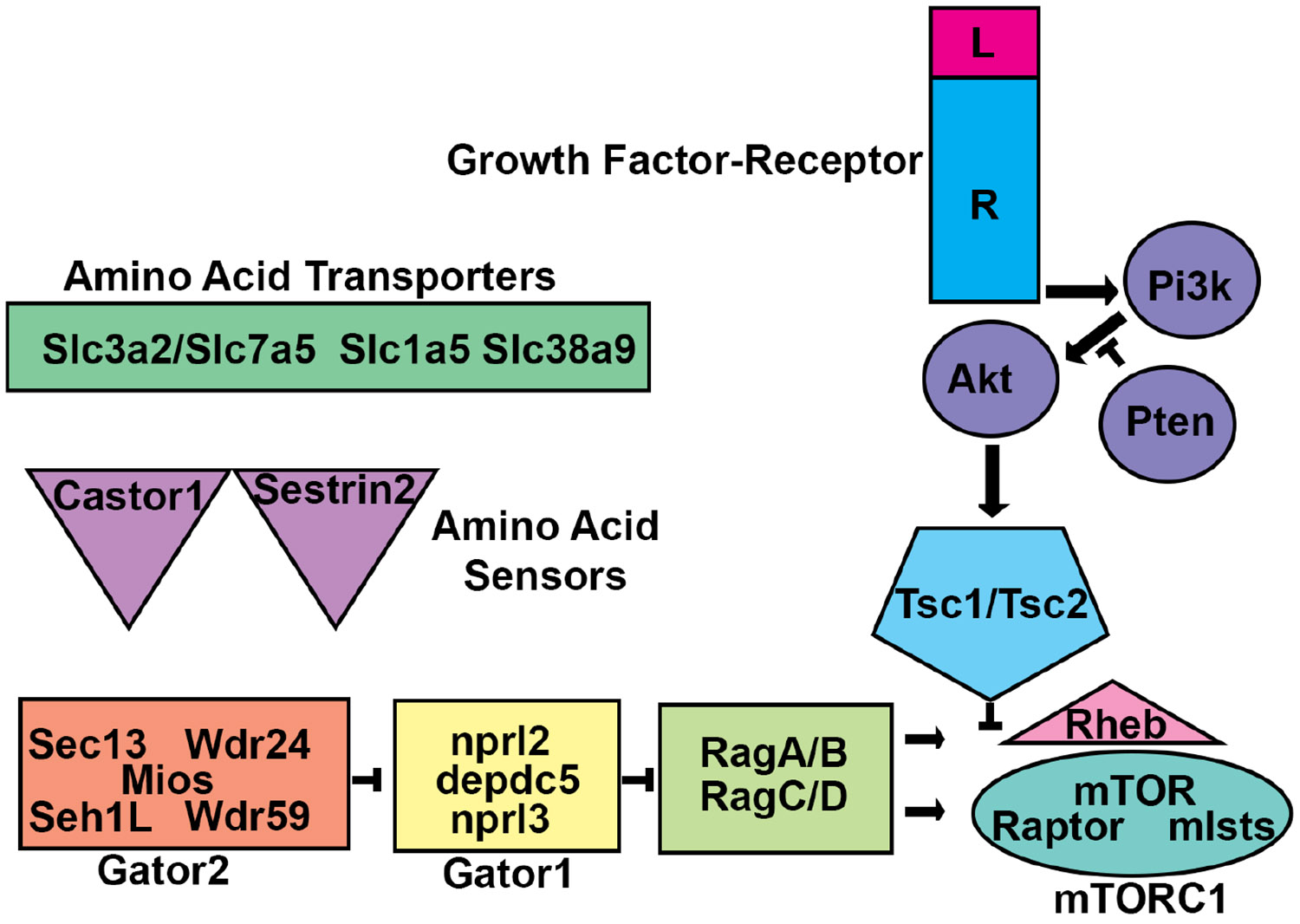
mTORC1 Regulation. Schematic diagram of the mTORC1 circuit. Amino acid transporters allow amino acids into cells and/or subcellular compartments. Amino acids are sensed by proteins such as castor or sestrin2. The sensor proteins then work through GATOR2 to prevent GATOR1, a GAP, from inhibiting the RAG heteromeric GTPases. RAG-GTP allows for mTOR Complex 1 (mTORC1) to interact with and become activated by the monomeric GTPase RHEB at the lysosome membrane. RHEB activation of mTORC1 is inhibited by the TSC1/TSC2 heteromeric GAP. TSC1/TSC2 can be inhibited by phosphorylation by protein kinases including AKT. AKT is activated by growth factor signaling initiated by transmembrane receptors which activate the lipid kinase PI3K. PI3K signaling is balanced by the lipid phosphatase PTEN. Mutations that alter these components can lead to malformations of cortical development.

A singular genome which is present within a fertilized zygote is responsible for generating the genomic and cellular architecture of an entire organism. In diploid organisms, half of the alleles are paternally derived, and half are maternally derived. When an allele is inherited, the frequency of each variant allele (VAF) is estimated to be ~50%. Genetic testing currently relies on this notion. For example, to determine the genetic cause of a disease, a patient may have DNA from blood, saliva, hair, or skin collected for genetic analysis. The expectation is that the patient has two inherited alleles with a VAF of 50% and deviations from this expectation may underlie disease. It is well accepted that only certain tissues or cell types may be changed in many diseases. One possible mechanism for why only specific tissues or cell types are affected in some diseases is that cellular genomes within an organism can change. Indeed, studies on Zea mays and later in patients having hemophilia demonstrated that cellular genomes may be altered by endogenous genomic modifiers called transposons (McCLINTOCK, 1950; Kazazian et al., 1988). Seminal studies on oncoviruses, oncogenes, and tumor suppressors also highlighted that the somatic genome was mutable (Varmus et al., 1972; Nigro et al., 1989; Ballester et al., 1990; Li et al., 1997; Kwon et al., 2006). Thus, postzygotic *de novo* mutations may cause two cells within an organism to be genetically different. This genetic mosaicism can have significant effects leading to neurological diseases, the causes of which have been undetectable until recently. Low VAF in regions of the brain are now realized to underlie some neurodevelopmental disorders which can be detected because of advances in DNA sequencing and computational analyses. Somatic genomic instability was also statistically modeled for sporadically arising diseases which were hypothesized to arise from *de novo* mutations (Knudson, 1971). This model, called the Two-hit hypothesis extends to numerous neurocutaneous disorders, including proteus syndrome with hypomelanosis of ito, for which patients have visually recognizable cutaneous somatic changes. Rudolf Happle argued that somatic mosaicism underlies the development of these disorders (Happle, 1987). Contemporaneously, evidence for somatic mosaicism was found in the growths of neurocutaneous syndromes Neurofibromatosis (NF) and Tuberous Sclerosis Complex (TSC) which are also characterized by neurocutaneous lesions and benign growths in the brain (Nigro et al., 1989; Verhoef et al., 1995). However, the role of somatic mutations, ranging from transposition events to point mutations, has only recently been recognized as a normal part of brain development (Bizzotto and Walsh, 2022). In some cases, somatic mosaicism that alters select pathways can cause malformations of cortical development that lead to a range of neurological manifestations and are the cause of numerous neurodevelopmental disorders. A fundamental neuroscientific question that remains then, is why such a mechanism for genomic heterogeneity exists. As more patients, tissues, and cells are sequenced and as sequencing technologies advance, the prevalence of somatic variants is sure to grow. Hopefully, so too will the appreciation for the capacity of variants to shape neurological function. This review will highlight the prevailing evidence that somatic mutations that alter mTOR function change neuronal circuitry and behavior and that this can be modeled in rodents which may be relevant for understanding the importance and role of genetic mosaicism in shaping normal physiological function. What follows is a description of the mTOR complex 1 pathway (Figure 1).

## Description of the components of the mTOR complex 1 Pathway

mTORC1 and mTORC2 are complexes that utilize the mechanistic target of rapamycin (mTOR) as a core catalytic kinase (Laplante and Sabatini, 2012). mTORC1 is inhibited by rapamycin, a molecule synthesized by *Streptomyces hygroscopicus* (Vézina et al., 1975). Rapamycin acutely inhibits mTORC1 but sustained exposure can also prevent the assembly of mTORC2 (Chung et al., 1992; Brown et al., 1994; Sabatini et al., 1994; Sabers et al., 1995; Burnett et al., 1998; Sarbassov et al., 2006; Lamming et al., 2012). Rapamycin works by causing FK506-binding protein (FKBP12) to bind to and inhibit mTORC1 (Sabatini et al., 1994). mTORCs contain homodimeric circular catalytic mTOR kinase loops held together by mTORC specific proteins (Yip et al., 2010; Aylett et al., 2016; Saxton and Sabatini, 2017). These proteins are Raptor and Rictor which stabilize mTORC1 and mTORC2, respectively (Hara et al., 2002; Dos et al., 2004).

mTORC1 is activated by the monomeric GTPase, RHEB (Garami et al., 2003; Inoki et al., 2003; Tee et al., 2003; Zhang et al., 2003). RHEB-GTP activates mTORC1. RHEB can hydrolyze GTP to GDP (Yamagata et al., 1994). When this happens, mTORC1 is turned off. RHEB dependent GTP hydrolysis is tightly controlled by hamartin and tuberin which are encoded by the *TSC1* and *TSC2* genes (Beugnet et al., 2003; Garami et al., 2003; Inoki et al., 2003; Zhang et al., 2003). Hamartin and tuberin form a GTPase activating protein (GAP) complex that prevents RHEB-mTORC1 activation. Tuberin exerts GAP activity towards RHEB. Hamartin stabilizes tuberin along with TBC1D7 (Dibble et al., 2012). mTORC1 activity is elevated in the absence of hamartin, tuberin, and TBC1D7. In addition, hamartin and tuberin are targeted by signaling pathways that titrate mTORC1 activity. For example, AKT phosphorylates and inhibits tuberin (Cai et al., 2006).

The amino acids leucine, arginine, and glutamine facilitate RHEB-mTORC1 activation (Hara et al., 1998). Activation is promoted by mTORC1 translocation to the lysosome where RHEB is located (Sancak et al., 2010; Bar-Peled et al., 2012; Efeyan et al., 2012). Translocation is mediated by heterodimeric RAG GTPases (RagA or B bound to RagC or D) (Bar-Peled et al., 2012). RAG-GTP facilitates interaction of and activates RHEB-MTORC1. RAG-RHEB-MTORC1 interaction is opposed by a GAP called GATOR1 (Bar-Peled et al., 2013). GATOR1 is comprised of DEPDC5, Nprl2, and Nprl3. GATOR1 turns off RAG-RHEB-mTORC1. A second complex called GATOR2 counters GATOR1 (Bar-Peled et al., 2013). GATOR2 (Mios, WDR24, WDR59, Seh1L, Sec13) inhibits GATOR1 thereby turning on RAG-RHEB-mTORC1. GATOR2 inhibits GATOR1 when leucine and arginine sensors are engaged and allow for RAG-RHEB-mTORC1 activation (Chantranupong et al., 2014; Chantranupong et al., 2016; Wolfson et al., 2016).

mTORC1 controls translation of select mRNAs by phosphorylating the eukaryotic initiation factor 4E (eIF4E) binding protein (4EBP), which is an inhibitor of eif4E (Burnett et al., 1998; Gingras et al., 1999). mTORC1 also phosphorylates p70S6 kinase (p70S6K) which catalyzes ribosomal S6 subunit (S6) phosphorylation (Chung et al., 1992; Price et al., 1992). mTORC1 dependent 4EBP inhibition and S6 activation stimulates translation of anabolic mRNAs involved in processes including ribosome biogenesis (Choo et al., 2008; Thoreen et al., 2012). mTOR also regulates catabolic cellular processes (Ganley et al., 2009; Jung et al., 2009; Kim et al., 2011; Zhang and Manning, 2015).

**FIGURE 2:**
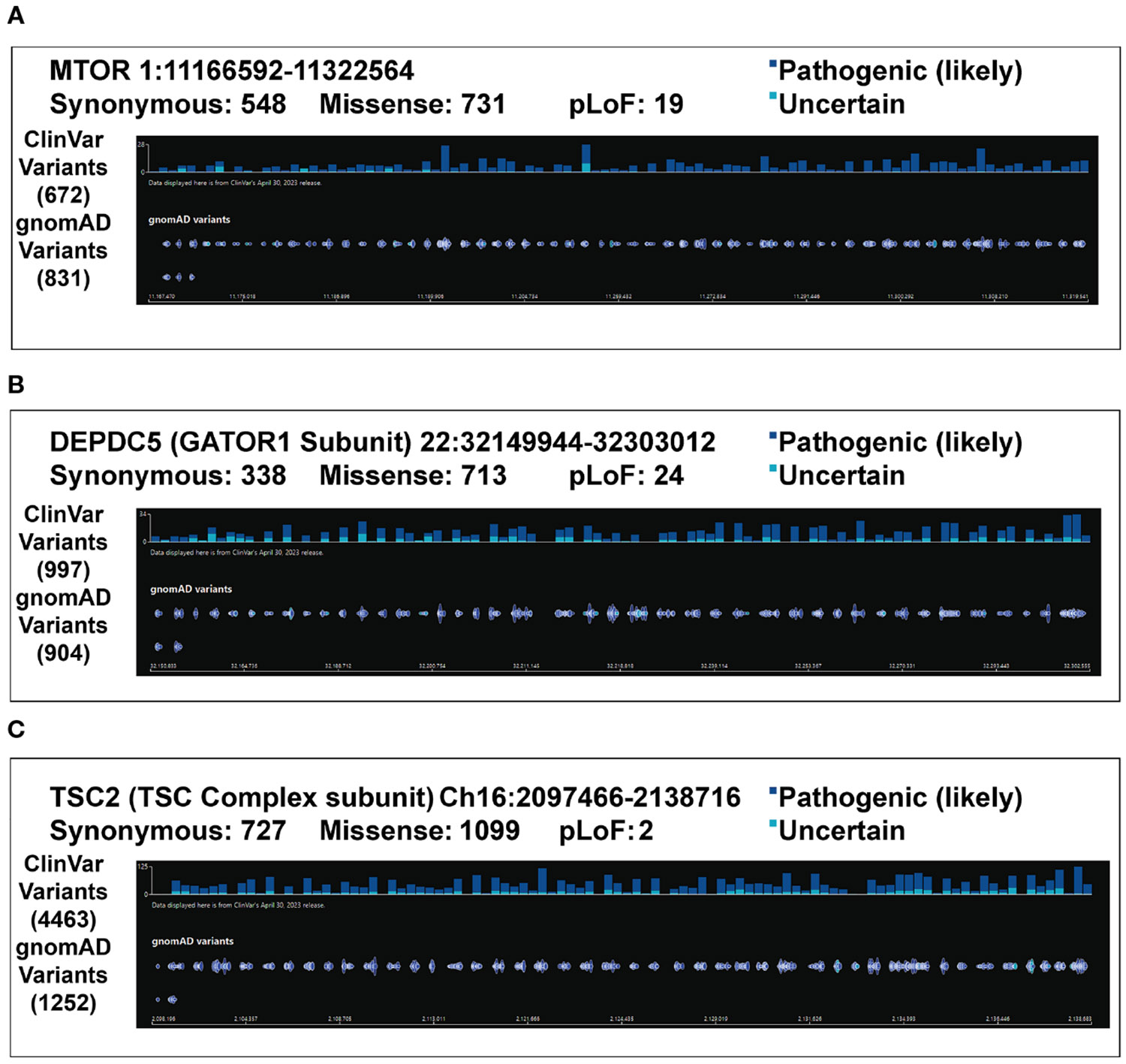
Pathogenic Variants. Variants of pathogenic or likely pathogenic significance as well as uncertain significance are listed for **(A)** *MTOR* **(B)** *DEPDC5* **(C)** *TSC2* from gnomAD browser (Karczewski et al., 2020). Note that both ClinVar variants and gnomAD variants are provided. Synonymous variants are not demonstrated due to space constraints. Despite a significant number of pathogenic variants for each gene that have been discovered, many more of uncertain significance have been identified and warrant further investigation.

Somatic mutations occurring along the mTOR pathway alter brain development. What follows is a description of case examples of mutations and the commensurate abnormalities that occur in the CNS. Next is a discussion of pathogenic variants organized by genes (Figure 2) and reported mutations (Table 1) starting from the catalytic subunit *MTOR*.

## Pathogenic variants that alter the mtorc1 pathway and cerebral cortical development

### MTOR variants and cortical development

A 5 year old patient having focal skin discoloration (hypomelanosis of ito) and hemimegalencephaly (HME) was discovered to harbor a somatic mutation in *MTOR* (c.4448C>T; p.Cys1483Tyr) at a 8-36% variant allele frequency (VAF) in tissue removed from affected cortex (Lee et al., 2012). HME is an enlargement of one side of the brain, typically involving the cerebral cortex and often contains improper lamination of the cortex with dysmorphic cytomegalic neurons. The patient had cortical dyslamination with ectopic and cytomegalic neurons. This patient had hypomelanosis of ito too which is interesting given the low allele frequency within the brain and the fact that these mutations were not found in any blood sample (Lee et al., 2012). These results suggest that the mutation might have arisen in cells that give rise to both the skin and cortex or that one of the inherited alleles is a mutant allele. It would be useful to know whether skin lesions also contained the same *MTOR* mutation. Nevertheless, this patient had elevated mTORC1 activity indicated by increased pS6 in brain tissue. Whether MTORC2 signaling was also elevated was not examined. This could be critical for developing potential patient therapies since rapalogs only target MTORC1. A second patient with HME and complex partial seizures was discovered that had a 14% VAF for p.Cys1483Tyr (D’Gama et al., 2015). D’Gama and colleagues subsequently reported 8 patients with mosaic MTOR mutations (D’Gama et al., 2017). The presence of this same mutation and another (*MTOR* c.5005G>T, p.A1669S) was later reported in a study examining focal cortical dysplasia (FCD) (Mirzaa et al., 2016). FCD is reminiscent of HME with the exception that FCD is confined to a smaller cortical region. FCD can be classified into type I, type IIa or type IIb and type III. FCDIIa and FCDIIb have focal mislamination, ectopically positioned cytomegalic, and dysmorphic neurons and differ based on the presence of balloon cells found in FCDIIb.

**FIGURE 3:**
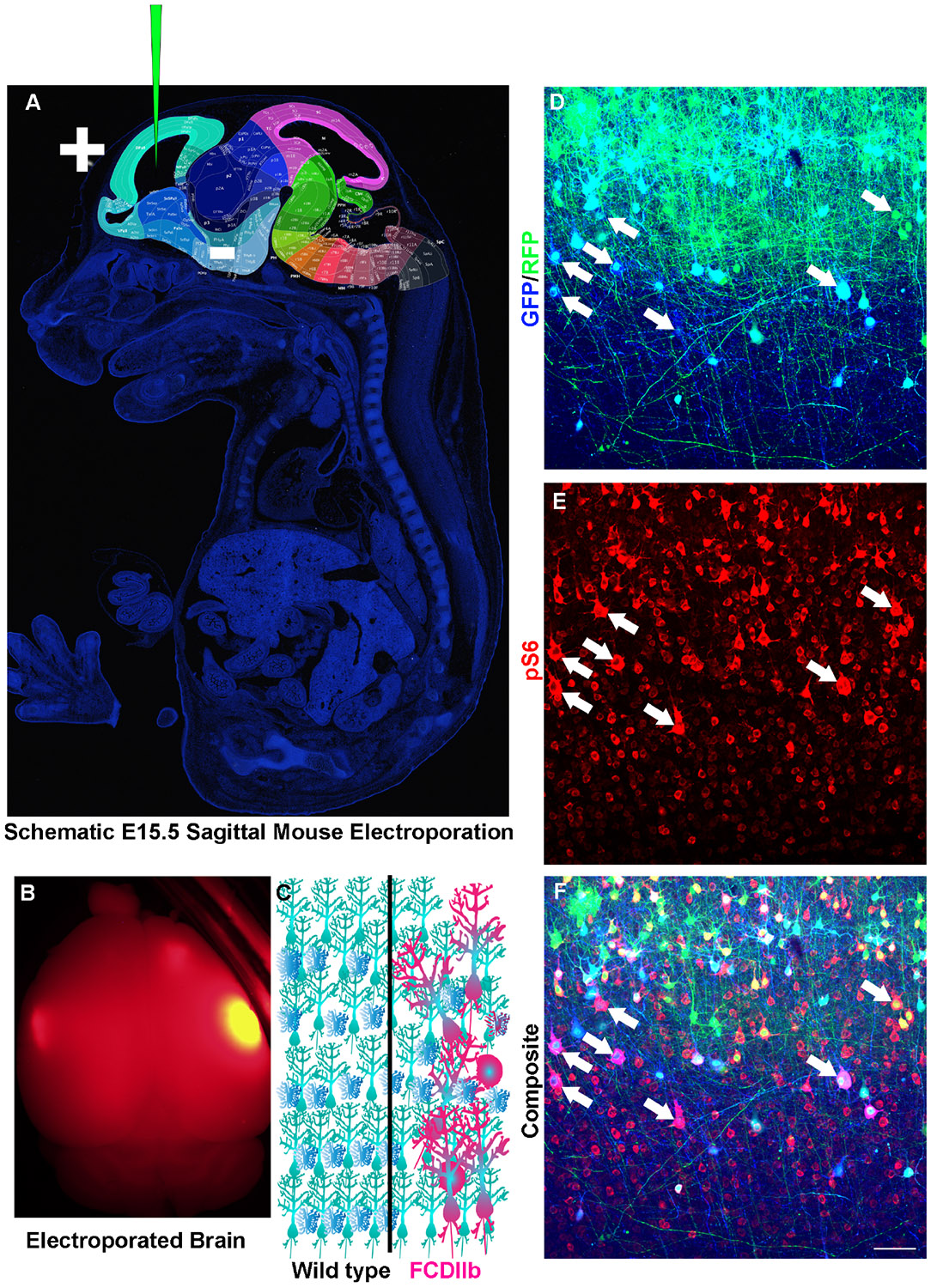
Modeling Somatic Mosaicism. **(A)** Sagittal section of an E15.5 mouse with regions of the brain highlighted and labeled. Modified from Allen Institute for Brain Science. Depicted is a glass capillary tube with plasmids (Green) inserted into the developing embryo. Injection of plasmid is followed by injection of short electrical pulses that facilitate the entry of plasmid into target cells. *In utero* electroporation as discussed here is typically targeted to radial glia which are neural stem cells that generate neurons and astrocytes. **(B)** An example of an adult mouse brain from an e15-15 electroporation. **(C)** Schematic diagram of wild-type (left) and Focal cortical dysplasia IIb (FCDIIb, right) brains. Note the cytomegalic and dysmorphic neurons along with balloon cells that are strewn throughout the normally hexalaminar cerebral cortex. **(D–F)** *In utero* electroporation of an e15-16 mouse with CRE recombinase and GFP (Blue) into RFP (Green) inducible mice having conditional and mutant TSC genes and stained for phospho-S6 (Red). Arrows point to cytomegalic neurons and balloon cells.

*MTOR* mutations were also found in ~15% of all patients diagnosed with FCDII (Lim et al., 2015). Over-expression of plasmids encoding representative MTOR mutations in HEK293T cells increased mTORC1 signaling which mirrored immunohistochemical quantification of mTORC1 activity in resected FCD. Several groups have introduced DNA plasmids into neural stem cells (NSCs) that generate the different types of cells in the brain using a technique called *in utero* electroporation (Figure 3). *In utero* electroporation of mutant *MTOR* plasmids into the developing cerebral cortex of mice induced rapamycin sensitive mTORC1 activity and seizures (Lim et al., 2015). These results demonstrate that the developing rodent cerebral cortex may allow for screening the veracity of claims that a somatic variant is pathogenic. A heroic study further examined 16 *MTOR* mutants in culture and performed *in utero* electroporations of select mutants having the greatest effects on MTORC1 pathway activity thereby demonstrating the utility of this methodological pipeline for assessing pathogenic variants (Tarkowski et al., 2019). Moreover, therapies may also be tested in these models.

VAF correlates with the clinical presentation of patients that have mTOR pathway mutations and cortical malformations. Patients with a low *MTOR* VAF have less severe malformations (FCD IIa) whereas those with a higher *MTOR* VAF had severe or diffuse HME (including with polymicrogyria) (Mirzaa et al., 2016). VAF or the presence of additional mutations was not examined in HME brain tissue which prevented direct comparisons, however. Nevertheless, tissue with greater changes to cytoarchitecture also had greater mTORC1 activity. Importantly, over-expression of mutant MTOR enhanced mTORC1 pathway activity and cell size in rat neurons which could be rescued with the mTORC1 inhibitor RAD001 (Mirzaa et al., 2016). Thus, lissencephalic mice and gyrencephalic rat brains both have neurons that can be altered by MTOR mutants and screened using drug therapies.

An additional 18 cases of somatic *MTOR* mutations were identified in a group of 76 out of 283 patients for which mutations were detectable (Chung et al., 2023). Although additional somatic mosaic MTOR mutations were identified and could phenocopy the FCD aspects of HME, why such changes occur was unclear (Hanai et al., 2017). *In utero* electroporation of plasmids encoding p.Cys1483Tyr and p.Leu-2427Pro mutations found in patients also causes abnormal development leading to FCD-like changes (Kim et al., 2019). Importantly, the rodent cortex again demonstrated utility for screening cellular and neuroanatomical changes caused by variants (Kim et al., 2019). FACS sorted neurons had translational changes associated with ribonucleoside metabolic pathways, RNA, processing, and regulation of organelle and cilia assembly (Kim et al., 2019). The link to ciliagenesis was solidified by the demonstration that activating *MTOR* mutations prevent autophagy causing a buildup of OFD1 (Park et al., 2018). OFD1 buildup prevented the generation of non-motile cilia and thereby abrogated WNT mediated morphological polarization (Park et al., 2018). These results indicate that reactivation of autophagy could be a useful treatment to treat patients. Rapamycin has a well-established role in activating autophagy in yeast and has some effect within mammalian brain (Noda and Ohsumi, 1998). However, other treatments, for example, those that activate pro-autophagic pathways such as AMPK activation of Ulk1 could also be useful (Egan et al., 2011). Moreover, autophagy plays a critical role in synaptic pruning (Tang et al., 2014). Altered mTOR pathway activity in ASD patients has revealed mTOR-dependent inhibition of autophagy may mediate hyperconnectivity (Tang et al., 2014). Not surprisingly, many patients with FCDs or HME also have ASDs. One surprising finding comes from a CRE-inducible transgenic mouse model harboring 4-point mutations in *MTOR* making it constitutively active (Kassai et al., 2014). This study utilized an *EMX1*-CRE mouse to turn on MTOR in excitatory neuron stem cells in the embryonic brain (Kassai et al., 2014). The result was massive cortical neuron apoptosis and microcephaly which is not reported in *in utero* electroporation models. However, assessing such effects following electroporation is a challenge since electroporation efficiency and success rate of electroporation are not easily assessable. It is however plausible that mutations originate in a different neural stem cell pool and produce different results. Alternatively, there may be limited nutrients or extracellular factors that limit the utility of using this global model. Nevertheless, the generation of a transgenic mouse demonstrates an interesting conundrum for electroporation. Plasmids are not genomically integrated at an efficient rate and therefore electroporation seldom allows for the consistent assessment on certain cell types. For example, astrocytes and neural stem cells are challenging to target using plasmid electroporation. However, the use of transposon technology during electroporation overcomes some of these limitations (Chen et al., 2015).

## RHEB variants and cortical development

MTORC1 must associate with RHEB-GTP to be activated. Electroporation of a constitutively active (CA) RHEB (S16H) mutant into neonatal and subsequently, embryonic mice, revealed phenotypes associated with hyperactivation of MTORC1 including MTORC1 pathway activation and ectopically positioned cytomegalic neurons (Yan et al., 2006; Lin et al., 2016). In utero electroporation of CA-RHEB led to seizures which could be initiated independent of lamination defects (Hsieh et al., 2016). Moreover, the amount of plasmid can be titrated to mimic the variable levels of MTORC1 activity that occur in patients (Nguyen et al., 2019). It could also be useful to vary promoters for electroporation studies since dilution of plasmids has resulted in a gradient of change which has not yet been quantified. Nevertheless, RHEB effects can be reversed by expressing a constitutively active form of the translation regulatory protein EIF4E-BP (Lin et al., 2016). These results indicated that *RHEB* mutations could theoretically cause cortical malformations. This hypothesis was soon realized in the discovery of patients with *RHEB* mutations that had intellectual delay and megalencephaly (Reijnders et al., 2017). Indeed, electroporation of mutant or wildtype RHEB expression is sufficient to induce most cellular phenotypes (Moon et al., 2015; Sokolov et al., 2018). A somatic *RHEB* mutation (c.119A > T: p. Glu40Val) was subsequently identified in an HME patient with an FCDIIb histopathology (Salinas et al., 2019). An additional FCD patient having somatic *RHEB* mutations in two adjacent nucleotides (A104T, C105A) resulted in a p.Y35L mutation that enhanced GTP binding (Zhao et al., 2019). *In utero* electroporation of this mutant into mice phenocopied previous mutant RHEB experiments and created another FCDII model. A similar *RHEB* p.Y37L mutant which produces stronger and *TSC1/2* resistant activation of MTORC1 activity in comparison to the *RHEB* S16H mutant also induces seizures that are independent of heterotopias that are formed (Onori et al., 2021). In addition to enhanced dendrite arbors, mutant neurons also have faster growing axons with extensive ramifications (Onori et al., 2021). Mutant axons had a broader and ectopic (lower layer) targeting in the contralateral hemisphere. Although the neurons had enhanced excitability, blockade of axon vesicle release could reverse the spontaneous seizures in this model (Onori et al., 2021). *RHEB* pY35L mutations have since been identified in other patients with FCDIIB and HME (Lee et al., 2021). Although *RHEB* mutations occur infrequently this work builds a critical foundation for understanding how somatic mutations that influence the MTORC1 p athway can cause cytoarchitectonic errors and lead to neurological manifestations including seizures (Chung et al., 2023). There are several major questions about *RHEB* mutations which exist. For example, are there mTORC1 independent effects? Another surprising fact is that mTOR requires signals to become localized to the lysosome. Do *RHEB* mutations only enhance the level of mTORC1 signaling or is the duration of mTORC1 signaling also increased? If the latter, how can this occur if the subcellular localization of MTORC1 is also under stringent control? Could this indicate that some amount of MTORC1 is always in proximity to *RHEB*, and that the level of RHEB-GTP is more important for MTORC1 activation than subcellular localization of MTORC1? Finally, does the subcellular distribution or kinetics of localization of mutant RHEB change? Answering these questions may help to generate better mechanistic insight into pathogenesis and to developing therapeutic strategies.

## TSC

RHEB is inhibited by a GAP comprised of proteins encoded by the *TSC1* and *TSC2* genes (Garami et al., 2003; Inoki et al., 2003; Zhang et al., 2003). Inactivating *TSC1/TSC2* mutations promote mTORC1 pathway activity (Feliciano, 2020). Inactivating mutations in *TSC1* or *TSC2* cause TSC (The European Chromosome 16 Tuberous Sclerosis Consortium, 1993; Van Slegtenhorst et al., 1997). TSC is a multi-system disorder characterized by prominent focal dysplastic regions including within the brain (Feliciano et al., 2013; Hasbani and Crino, 2018; Feliciano, 2020). The majority of TSC patients have seizures (Kingswood et al., 2014; Kingswood et al., 2017; Nabbout et al., 2019). Seizures called infantile spasm frequently occur in infants with focal seizures developing later on (Lux and Osborne, 2004; Kelley and Knupp, 2018; Nabbout et al., 2019). Seizure manifestations are heterogenous (Nabbout et al., 2019). Seizures are likely caused by focal dysplastic regions called cortical tubers that are present in the majority of patients (Krueger et al., 2013b; Kingswood et al., 2017). Seizure foci often overlap with cortical tubers (Mohamed et al., 2012). Electrophysiological analysis of tubers demonstrated that tubers have an extensive contribution to cortical hyperexcitability (Despouy et al., 2019). In agreement, cortical tuber removal prevents seizures (Fallah et al., 2013; Fallah et al., 2015). Surgical removal of peri-tuber regions enhances outcomes which indicates that regions outside of the tuber may also contribute to hyperexcitability (Fallah et al., 2015). Cellular and molecular changes within and surrounding cortical tubers may influence their electrophysiological properties, spread of hyperexcitability, and clinical presentation (Doherty et al., 2005; Boer et al., 2008; Kaczorowska et al., 2011; Canevini et al., 2018; Nabbout et al., 2019). There are however case reports of TSC patients without tubers that exhibit seizures which indicates additional mechanisms of epileptogenesis may exist in TSC (Wang et al., 2007). Moreover, removal of conditional *Tsc1* from postmitotic neurons in mice induces seizures in the absence of some tuber features and supports this hypothesis (Meikle et al., 2007; Wang et al., 2007). Tubers are histologically heterogenous (Chu-Shore et al., 2009; Gallagher et al., 2010; Zhang et al., 2018). They can vary in cellular composition (Mühlebner et al., 2016). Similar to FCD, tubers are regions for which the cortex loses laminar cytoarchitecture and have dysmorphic cytomegalic neurons and balloon cells (Ferrer et al., 1984; Huttenlocher and Heydemann, 1984; Mühlebner et al., 2016). However, tubers contain giant-cells (Yamanouchi et al., 1997a; Yamanouchi et al., 1997b; Mizuguchi and Takashima, 2001; Mizuguchi et al., 2002).

### Genetic mosaicism in TSC

Somatic mosaicism has long been recognized in TSC (Verhoef et al., 1995; Verhoef et al., 1999; Sampson et al., 1997). There are multiple types of mosaicism that can occur. It is important to keep in context how this might be relevant to defining which variants are pathogenic in the related disorders discussed in this review. The first type of mosaicism is germline mosaicism (Dabora et al., 2001). This occurs when a non-carrier parent produces gametes that may carry a mutation that is subsequently inherited. The second is classical mosaicism, which occurs when a *de novo* mutation arises during development (Verhoef et al., 1999; Qin et al., 2010b; Tyburczy et al., 2015; Lim et al., 2017; Klonowska et al., 2023). Although it is feasible to ascertain whether a parent carries a variant, it is challenging to determine the precise time of development when the *de novo* variant arises. However, groups have made compelling progress in this arena which might be clinically applicable (Bizzotto et al., 2021). Nevertheless, a late occurring mutation would affect a minor fraction of cells. The third type of mosaicism is that which Knudson proposed, which is the manifestation of the two-hit hypothesis (Knudson, 1971). In this model, a mutant allele and wild-type allele are inherited, followed by conversion of the wild-type allele into a mutant allele. Knudson later used the Eker rat, which carries a *Tsc2* mutation, to test this model (Eker and Mossige, 1961). They demonstrated that the tumors have *Tsc2* loss of heterozygosity (LOH) (Hino et al., 1993; Yeung et al., 1994; Kubo et al., 1995). While most TSC phenotypes require LOH, there are many reports that have used heterozygous rodents to suggest haploinsufficiency. One question not yet answered is how often these models have LOH. Thus, routine examination of tissue from heterozygous models should also examine conversion of the second allele.

### Cell culture models of TSC

A human induced pluripotent stem cell (IPSC) culture model was created that has conditional *TSC1* alleles to determine the effect of losing TSC genes on neurodevelopment(Blair et al., 2018). IPSCs were used to generate NSCs that were subsequently differentiated. Loss of both *TSC1* alleles was required for cortical tuber phenotypes (Blair et al., 2018). Winden et al. however demonstrated that heterozygous and homozygous *TSC2* mutant human IPSC derived NSCs exhibit significant changes that mirrored the conditional *TSC1* NSC model. Surprisingly, heterozygous and homozygous *TSC2* mutant *NSCs* had the same amount of tuberin (c.5238_5255del p.H1746_R1751del, *TSC2+/−*) (Winden et al., 2019). The difference between homozygous and heterozygous requirements in these models could be caused by the mutation, NSC type, culture condition, or LOH in the *TSC2+/−* cells. Detection of LOH in patient tubers while detectable, may be limited by VAF detection in low abundance cell types (Crino et al., 2010; Qin et al., 2010a). On the other hand, either mechanism may be sufficient to generate tubers depending on the mutation. Another human IPSC-NSC model demonstrated that *TSC2* mutant heterozygous IPSCs undergo LOH during culture (Eichmüller et al., 2022). These analyses challenged the proposition for LOH but were specific for a specialized caudal late inhibitory progenitor (CLIP) which has low tuberin expression. SEGAs and tubers are not symmetrically (bilaterally) localized in patients. Since both brain hemispheres have CLIP cells and both hemispheres would be proposed to harbor the same pathogenic variant, why only 5–20% of TSC patients develop SEGAs is unclear. Moreover, TSC patients’ cortical tubers are present in anatomically and cellularly distinct regions. In fact, TSC patients have many types of lesions outside of the brain which seldom occur symmetrically. In contradiction to a cell-type specific function of TSC genes, loss of both copies of *Tsc1* or *Tsc2* in many different neural cell types causes overlapping phenotypes in rodents. Finally, as discussed below, transgenic mice with conditional alleles subject to electroporation or crossed to CRE driver mice have more severe manifestations than heterozygous models. Nevertheless, the specificity of SEGAs occurring most frequently along the lateral ventricles near the foramen of Monroe is compelling. It is also of interest to consider that while no differential methylation pattern is currently recognized for maternal vs. paternal *TSC1* or *TSC2*, there may be additional mechanisms that factor into the penetrance of phenotypes.

### Mouse models of TSC

An inherited homozygous dominant negative mutation or two recessive mutations appear incompatible with mammalian viability. No cases of TSC patients that have inherited two mutant alleles has been reported. This is supported by the fact that *Tsc1* and *Tsc2* homozygous deletion is embryonic lethal in mice and rats (Rennebeck et al., 1998; Onda et al., 1999; Kobayashi et al., 2001). Moreover, *Tsc2* heterozygous neuroepithelial cells have no differences whereas homozygous null cells recapitulate key aspects of those seen in TSC (Onda et al., 2002). Mice having conditional TSC alleles have circumvented this limitation and demonstrated that phenotypes most often can be detected following loss of both TSC alelles (Kwiatkowski, 2002; Hernandez et al., 2007). For example, homozygous *Tsc1* deletion from NSCs generates mislamination, macrocephaly, cytomegaly, hypomyelination, and reactive gliosis with seizures (Goto et al., 2011; Magri et al., 2011; Carson et al., 2012; Mietzsch et al., 2013). Homozygous *Tsc2* deletion also generates macrocephalic mice having cytomegaly, hypomyelination, reactive gliosis and seizures (Way et al., 2009; Mietzsch et al., 2013). *Tsc1* removal from neurons using synapsin I promoter driven CRE also caused mTORC1 hyperactivation, cytomegalic and dysmorphic neurons, megalencephaly, severe seizures and premature mortality (Meikle et al., 2007; Wang et al., 2007). The absence of a discrete cortical tuber in TSC conditional mice has provoked discussions that loss of TSC genes in large cellular populations in the cortex might cause the entire cortex to represent a cortical tuber (Wong, 2012). To recapitulate LOH and the focal nature of cortical tubers, in utero electroporation of CRE recombinase into mice carrying a mutant and conditional *Tsc1* allele was performed (Feliciano et al., 2011). The *in utero* model generated focal mosaic patterning, cytomegaly, and hyper-active mTORC1 similar to that seen in patient tubers (Feliciano et al., 2011). Two conditional *Tsc1* alleles appears sufficient to allow modeling of this phenotype (Robens et al., 2016; Cox et al., 2018). In utero *Tsc1/2* knockdown also increased the number of axons in developing cortical neurons (Choi et al., 2008). Importantly, recent reports suggest that loss of *TSC* genes through a Two Hit mechanism can also cause FCD (D’Gama et al., 2017; Lim et al., 2017; Baldassari et al., 2019b; Jha et al., 2022; Chung et al., 2023). *In utero* electroporation of CRISPR-Cas9 system targeting TSC genes was used to model the FCD which produced cortical dyslamination, cytomegalic neurons, and induced seizures (Lim et al., 2017).Taken together, TSC1/TSC2 somatic mutations within TSC or FCD patients can cause malformations in the cortex that often underlie seizures. It remains to be seen whether such somatic mutations underlie the many other neuropsychiatric manifestations in these patients.

### Pharmacology of mTOR inhibitors TSC

The role of mTORC1 in the pathogenesis and clinical manifestations in TSC is underscored by the utility of clinically approved mTORC1 inhibitors. mTORC1 inhibitors include everolimus and sirolimous which are derived from the macrolide antifungal compound rapamycin which is synthesized by the bacteria *Streptomyces hygroscopicus* (Vézina et al., 1975). Rapamycin causes FK506-binding protein (FKBP 12) to inhibit mTORC 1 phosphorylation of select substrates including the p70 ribosomal S6 kinase (Chung et al., 1992; Brown et al., 1994; Sabatini et al., 1994; Sabers et al., 1995). mTORC1 phosphorylation of other substrates such as Ulk1 and 4EBP is however, incompletely inhibited by rapamycin (Choo et al., 2008; Thoreen et al., 2009; Kang et al., 2013). Despite these critical limitations, rapamycin analogs (rapalogs) are effective in treating TSC and FCD models. Rapamycin treatment of the *Synapsin*-CRE x *Tsc1* model reverses elevated mTORC1 pathway activity, neuron size, cortical thickness, and increased survival (Meikle et al., 2008). Similar results were achieved in NSC *Tsc1* deletion models (Anderl et al., 2011; Magri et al., 2011). The importance of mTORC1 in lesion formation was confirmed in focal models which revealed specific windows of time for which rapamycin reverses select cellular phenotypes (Tsai et al., 2014; Lim et al., 2017; Cox et al., 2018). SEGAs are TSC associated subcortical growths that occur within the brain of TSC patients. The first study to use mTORC1 inhibitors to treat these growths found that rapamycin was highly effective and caused SEGAs to regress (Franz et al., 2006). Everolimus treatment caused TSC SEGA regression by 30-50% within 6 months and coincidentally reduced seizure burden (Krueger et al., 2013a). 17 of 20 patients had a reduction in seizures with a 73% median decrease (Krueger et al., 2013c). Extension of the EXIST (EXamining everolimus In a Study of TSC) phase III clinical trials demonstrated long-term everolimus decreased seizure frequency and was particularly impressive at a high 9-15 ng/mL exposure (Curatolo et al., 2018). Early MRI and EEG fingerprints predict neurological outcome in TSC children with severity and age of seizure onset being a predictor of intellectual disability (De Ridder et al., 2021; Hulshof et al., 2022). Moreover, many neonates that have normal EEG before 2 months of age develop aberrant activity later and can represent an opportunity to prevent encephalopathy (Kingswood et al., 2014; Słowiń ska et al., 2018; De Ridder et al., 2021). Indeed, early intervention with vigabatrin improves neurological outcome with adjuvant everolimus further reducing seizures (Curatolo et al., 2018; Nabbout et al., 2021; Śmiałek et al., 2023). These results indicate that early treatment may facilitate proper brain development which could have lifelong implications and sets the stage for treating patients that have other mTORC1 pathway variants that cause cortical malformations.

## GATOR1

Amino acids facilitate RHEB-mTORC1 activation (Hara et al., 1998). Amino acids allow GATOR2 to inhibit GATOR1 (Bar-Peled et al., 2013; Parmigiani et al., 2014; Chantranupong et al., 2016; Kimball et al., 2016). GATOR1 is the GAP for the heterodimeric RAG GTPase (RagA or B bound to RagC or D) at the lysosome (Bar-Peled et al., 2013). Mutations in the GATOR1 components, *DEPDC5, NPRL2*, and *NPRL3* cause cortical malformations. Two seminal genetics studies were published in 2013.

### DEPDC5 variants and cortical development

One study described 19 families with autosomal dominant focal epilepsy were used for linkage analysis and identified 22q12 association Familial *DEPDC5* mutations were initially described as a cause of familial focal epilepsy with multiple foci. Further sequencing confirmed mutations including nonsense and missense mutations demonstrating loss of function of *DEPDC5* as the likely cause (Ishida et al., 2013). The other study demonstrated that *DEPDC5* mutations occur in >10% of families with non-lesional focal epilepsy (Dibbens et al., 2013). Patients from these families can have focal dysplasias (bottom of the sulcus) or focal band heterotopia (Scheffer et al., 2014). To provide mechanistic studies on *DEPDC5*, knockout rats were generated, but died embryonically (Marsan et al., 2016). Heterozygous *DEPDC5* rats still had dysmorphic cytomegalic neurons with pS6 and reduced firing rates, but do not exhibit seizures (Marsan et al., 2016). *DEPDC5* heterozygous e12.5 cultured neurons also have similar morphological changes (De Fusco et al., 2020). These results demonstrate that loss of a single allele could be sufficient for cellular phenotypes. However, the heterozygous mouse neurons do not have detectably higher pS6 *in vitro* as measured by western blot (De Fusco et al., 2020). In contrast, *DEPDC5* null e12.5 mouse neuron cultures as well as *DEPDC5* knockdown neurons have a more severe phenotype than heterozygotes including increased mTORC1 pathway activity and enhanced dendrite arbors (De Fusco et al., 2020). A requirement for loss of both alleles is consistent with a genome engineering approach using CRISPR/Cas9 and Talen technology to remove *DEPDC5* from mice (Hughes et al., 2017). Thus, one might consider Knudson’s two hit hypothesis as a potential mechanism for *DEPDC5* phenotypes. Indeed, using matched blood/brain lesion samples, Knudson’s two hit hypothesis again came to fruition (Baulac et al., 2015; Baldassari et al., 2019b). This result was further solidified by in utero electroporation of *DEPDC5* targeted gRNAs using CRISPR/Cas9 (Ribierre et al., 2018). Interestingly, these mice also develop seizures. In agreement, *in utero* electroporation and mutation of rat *DEPDC5* recapitulates this phenotype (Hu et al., 2018). However, a neuron selective CRE driver (synapsin-I promoter) mouse crossed to those having conditional *DEPDC5* exhibited nearly all phenotypes of patients (Yuskaitis et al., 2018). Mice also had spontaneous seizures associated with premature mortality which could be rescued by rapamycin (Yuskaitis et al., 2019). *Depdc5* conditional mice were crossed to *Depdc5* mice and subject to *in utero* electroporation of CRE recombinase, similar to the *Tsc1* tuber model, which was sufficient to model nearly all FCD phenotypes seen in FCD patients with *DEPDC5* mutations (Dawson et al., 2020). Taken together, it appears that loss of both alleles of *DEPDC5* can recapitulate the phenomenon seen in patients.

### NPRL3 and NPRL2 variants and cortical development

Linkage analysis of first cousins with focal epilepsy and later a cohort of FCD patients associated mutations in *NPRL3* with seizures (Sim et al., 2016). Another 5 mutations in *NPRL3* and 5 in *NPRL2* were identified in a cohort of 404 focal epilepsy patients (Ricos et al., 2016). Interestingly, the penetrance of mutations appeared low for some families (Korenke et al., 2016). Additional mutations in *NPRL2* and *NPRL3* continue to be identified in a range of epilepsy patients (Baldassari et al., 2019a). Using *Emx1-CRE* to drive recombination in the dorsal telencephalon led to a nearly overlapping phenotype of *Nprl3* and or *Nprl2* conditional mice (Ishida et al., 2022). Importantly, the same manuscript compared both models as well as conditional *Depdc5* mice (Ishida et al., 2022). These results confirmed that loss of both alleles of any GATOR1 component is sufficient to cause hyperactivation of the mTORC1 pathway and induce seizures (Ishida et al., 2022). The effect of *Nprl3* on neuron mTORC1 activity and neuron morphology was further validated using *in utero* electroporation CRISPR/Cas9 mediated mutation (Ishida et al., 2022).

## AKT variants and cortical development

The presence of PIP3 recruits AKT to the cell membrane where it is phosphorylated by phosphoinositide-dependent protein kinase 1 (PDK1) T308 (Alessi et al., 1997). Subsequent phosphorylation of AKT T473 leads to complete activation (Sarbassov et al., 2005). AKT phosphorylates numerous substrates that regulate cell survival (BAD), metabolism (GSK3a/b), and proliferation (p27 Kip1 and p21 Cip1). AKT is highly expressed within the developing brain (Owada et al., 1997). It is highly expressed in a proportion of embryonic NSCs and over-expression increases NSC proliferation including in the V-SVZ and OB (Sinor and Lillien, 2004).

Tuberin is a substrate of AKT (Cai et al., 2006). Tuberin along with hamartin, which are encoded by *TSC1* and *TSC2*, form a GTPase activating protein (GAP) that inhibit RHEB (Garami et al., 2003; Inoki et al., 2003; Tee et al., 2003; Zhang et al., 2003). Tuberin has a GAP domain that causes the monomeric GTPase RHEB to hydrolyze GTP (Tee et al., 2003). Hamartin and TBC1D7 together appear to stabilize Tuberin (Dibble et al., 2012). RHEB activates mammalian target of rapamycin mTOR (Garami et al., 2003; Inoki et al., 2003; Tee et al., 2003; Zhang et al., 2003). mTOR can also phosphorylate AKT 473. Specifically, an mTOR heteromer called mTOR Complex 2 that associates with a protein called rictor phosphorylates AKT phosphorylation (Sarbassov et al., 2005). Rheb activates a distinct mTOR complex (mTORC1) that contains the protein raptor (Hara et al., 2002; Kim et al., 2002; Dos et al., 2004).

Somatic *AKT1* mutations had been uncovered in proteus syndrome (Lindhurst et al., 2011). However, examination of *AKT1* in 20 patients with HME failed to reveal somatic mutations. However, somatic mutations in *AKT3* were discovered (Lee et al., 2012). A contemporaneous report of an *AKT3* mutation in an HME patient was described by another group that indicated *AKT3* activation normally occurs within dividing apical NSCs near the lumen of the ventricles (Poduri et al., 2012). However, an *AKT1* E17K pathogenic mutation was identified in a patient with HME and Proteous syndrome (D’Gama et al., 2017). *AKT3* E17K mutations were also identified in another HME patient in the same study (D’Gama et al., 2017). The recurrence of the AKT3 E17K mutation in studies of HME and FCD or related syndromes is exceptionally impressive and demonstrates the pathogenicity of this mutation (Baek et al., 2015; Jansen et al., 2015; Alcantara et al., 2017; D’Gama et al., 2017; Baldassari et al., 2019b). *In utero* electroporation of a plasmid encoding an *AKT3* E17K mutant which mimicked a mutation found HME patients, prevented excitatory cortical neuron lamination leading to ectopic positioning, dysmorphic phenotypes, and cytomegaly (Baek et al., 2015). Interestingly, AKT3 constitutive activity altered transcriptomes leading to expression of reelin which was responsible for luring and entrapping non-electroporated cells. Somatic duplication of a region containing *AKT3* have also been reported (Conti et al., 2015). Taken together, *AKT3* mutations are a cause of abnormal cortical development. It is unclear to what extent the abnormal activity of *AKT3* during development as opposed to after development, within postmitotic neurons causes neurological manifestations such as epilepsy. It would appear however, that hyperactivation caused by mutations to *AKT3* or upstream regulators of *AKT3* could be treated with *AKT3* inhibitors and is of further clinical exploration. Given that AKT3 is a protein kinase, a major question that remains is which substrates are responsible for the changes seen in patients with AKT. The AKT substrate that rises above all others is tuberin, the inactivation of which causes a range of neurological manifestations.

## PI3K

### PIK3CA variants and cortical development variants and cortical development

Information conveyed by growth factors are transmitted by receptors having tyrosine kinase activity. The ability of somatic alterations that effect integral membrane proteins to change cells was demonstrated by oncogenic viruses that induce cellular transformation (Frykberg et al., 1983; Downward et al., 1984). Such growth factor receptor kinases exert effects by acting on intracellular signaling proteins. Later it was demonstrated that an avian sarcoma virus that encoded oncoviral proteins had similar transforming activity but acts as an intracellular signaling kinase (Macara et al., 1984; Sugimoto et al., 1984; Whitman et al., 1985; Kaplan et al., 1986). This transforming activity was identified to be mediated by a lipid kinase (Whitman et al., 1988). However, this lipid kinase phosphorylated the 3-OH group of phosphatidylinositols and generated phosphatidylinositol (3,4,5)-trisphosphate (PIP3) (Auger et al., 1989; Ruderman et al., 1990). PIP3 subsequently acts as a second messenger to help recruit and activate AKT. The oncoviral studies support the idea that somatic changes in lipid signaling can also alter the balance of cell division. A hint that mutations in PI3K could disrupt brain development came from studies of a girl that had megalencephaly-polymicrogyria-polydactyly-hydrocephalus syndrome (MPPH) which revealed a mutation in *PIK3R2* (Nakamura et al., 2014). The *PIK3R2* mutation was detected in blood leukocytes at a VAF of 47.7%. LOH or de novo mutations within the brain were not examined, but the authors concluded based on VAF, that the mutation was likely a *de novo* germline mutation (Nakamura et al., 2014). The first report that somatic mutations may result in hemimegalencephaly included identification of several patients have *PI3KCA* mutations. One mutation, *PIK3CA* c.1633G>A (p.Glu545Lys) was recurrent (Lee et al., 2012). This mutation was also identified by other groups (D’Gama et al., 2015; D’Gama et al., 2017; Baldassari et al., 2019b). However, a different mutation, PIK3CA pH1047R which is associated with diverse cancers inspired D’Gama and colleagues to cross a previously generated inducible form of this mutation to different CRE driver mice. EMX1-CRE dependent expression of the PIK3CA mutant caused MEG, abnormal cortical gyrification, and abnormal lamination thereby recapitulating many HME/FCD-like phenotypes (D’Gama et al., 2017). A larger study later built off of this finding and uncovered additional patients with *PIK3CA* mutations that had HME (Chung et al., 2023). Single cell RNA sequencing of two of these patients uncovered changes in the cellular composition and revealed altered transcript abundance that indicated altered lipid biosynthesis and metabolism in astrocytes.

### PIK3R2 and PIK3R3 variants and cortical development

Somatic mutations in *PIK3R2* encoding regulatory subunit of PI3K have also been discovered in numerous patients. Notably, mosaic mutations in children with bilateral perisylvian polymicrogyria (Mirzaa et al., 2015). This mutation was further modeled in mice which had extensive megencephaly, mild ectopic neurons, cytomegaly, increased pS6 and EEG seizures (Shi et al., 2020). In addition, PI3K regulatory subunit (*PIK3R3*) mutations have also been described (Chung et al., 2023). Taken together, somatic mutations that increase PI3K activity, also disturb brain development. These studies generate many provocative questions, namely whether there are convergent mechanisms by which PI3K is working, could PI3K inhibition be used to treat these patients, is it a specific lipid product such as PIP3 that is altering brain development or a non-physiological product at fault, and finally, do all cells affected by these mutations cause the anatomical and clinical presentations or is there any specificity of how the mutations change brain development.

## PTEN variants and cortical development

Mechanisms for reducing PIP3 also exist to prevent excessive downstream signaling. Evidence of such a system came from studies on somatic mutations that occur in brain tumors. LOH on chromosome ten q23 encoding a lipid phosphatase with homology to chicken tensin, and therefore called *PTEN*, is a common occurrence in cancers including glioblastoma (Li et al., 1997). *PTEN* is a lipid phosphatase, and like tensin, has a Src Homology 2 (SH2) domain allows for docking at phospho-tyrosine residues. *PTEN* removes the 3’ phosphate of the inositol ring of phosphatidylinositol (3,4,5)-trisphosphate (PIP3), resulting in phosphatidylinositol (4, 5)-bisphosphate (PIP2) (Parsons, 2020). Inactivating mutations in PTEN therefore lead to a buildup of PIP3, a special lipid messenger. *PTEN* appears to function as a tumor suppressor, as its loss is readily detected in glioblastoma. Bannayan-Riley-Ruvalcaba syndrome, Cowden syndrome, multiple hamartoma syndrome, and proteus-like syndrome are now covered by the umbrella term *PTEN* Hamartoma syndrome (Parsons, 2020).

Based on the importance of growth factor receptors in NSCs, it is not surprising that conditional deletion of *PTEN* using a nestin promoter CRE enhanced proliferation of tripotent neural progenitors (Groszer et al., 2001). Likewise, loss of *PTEN* from e14.5 NSCs enhances neurosphere self-renewal and cell cycle reentry (Groszer et al., 2006). Removal of *PTEN* using GFAP-CRE which is expressed in NSCs causes abnormal cytoarchitecture including in the hippocampal dentate gyrus where NSCs persist (Backman et al., 2001; Kwon et al., 2001). But many of the alterations are due to changes in neurons born from NSCs where *PTEN* loss increases cell size. Regardless, *PTEN* deletion in the adult V-SVZ also enhances neurogenesis (Gregorian et al., 2009). Likewise, removal of *PTEN* from the postnatal V-SVZ using tamoxifen inducible CRE expressed in nestin positive NSCs increases the number of neuroblasts in the V-SVZ. *PTEN* null neuroblasts likely have cell autonomous precocious differentiation since ectopically positioned neurons were present along the rostral migratory stream and in the SVZ (Zhu et al., 2012) *PTEN* deletion from human progenitors also alters cerebral organoids, causing a transient delay in differentiation, and promoting increased organoid size and gyrification (Li et al., 2017).

In combination with loss of other tumor suppressors such as *NF1* and *p53, PTEN* deletion induces glioblastoma in mice (Llaguno et al., 2008). Interestingly, as reported for NF1 heterozygous mice, combined *NF1/PTEN* heterozygous deletion is insufficient for tumor formation (Llaguno et al., 2008). In contrast removal of one *PTEN* allele with both *p53* alleles causes highly penetrant astrocytoma (Zheng et al., 2008). Whether LOH of *PTEN* occurred in this manuscript was unclear. Nevertheless, these studies point to the fact that the NSCs collected from the V-SVZ are likely tripotent progenitors since they have the capacity to generate neurons, astrocytes, and oligodendrocytes. These results could mean, that some NSC populations could be more susceptible to oncogenic transformation than others. In agreement, *PTEN* deletion in astrocytes appeared to have minimal effects on cytomegaly, proliferation, or activation of downstream pathways but combined *PTEN* and *p53* deletion generated tumors that more frequently associated with the V-SVZ (Chow et al., 2011). *PTEN* deletion along with constitutive activation of PI3K does however cause high grade brain tumors that protrude from the V-SVZ into the lateral ventricles (Daniel et al., 2018). On the other hand, expression of constitutively active PI3K is sufficient to generate oligodendrogliomas indicating that there may be additional lipid phosphatases that might substitute for *PTEN* or that insufficient PIP3 builds up to form tumors in *PTEN* null NSCs (Daniel et al., 2018).

*PTEN* deletion also alters neuron morphology throughout the brain. One particularly striking example is that *PTEN* deletion using an *NSE-CRE* driver caused widespread cortical and hippocampal alterations (Kwon et al., 2006). These animals were macrocephalic, had neuron cytomegaly, and increased dendrite arborization and axon growth with enhanced synaptogenesis. These animals also revealed sensory hyperactivity with altered social responses. Neuron morphology is reproducibly altered in cortical pyramidal neurons (Chow et al., 2009). *In utero* electroporation of conditional *PTEN* mice has also confirmed a prominent role in regulating cortical neuron dendrite growth (Hsia et al., 2014). The use of CRISPR/Cas9 to remove *PTEN* has also confirmed that cortical neurons have cytomegaly and revealed that these neurons have altered excitatory post-synaptic currents (Chen et al., 2015).

Somatic mutations in *PTEN* are widespread in brain tumors. In addition, mutations are reported in a range of non-cancer syndromes which include Cowden Syndrome, Lhermitte-Dulcos disease (cerebellar dysplastic gangliocytoma), Bannayan-Riley-Ruvalcaba syndrome (BRRS), and autism spectrum disorder with macrocephaly. Cerebellar dysplastic gangliocytoma are dysplastic growths within the cerebellum. The prevalence of somatic mosaicism or LOH in these lesions is largely unknown, however most have inherited at least one mutant allele and at least one study has detected LOH (Zhou et al., 2003). One notable example was reported by Janesen et al. whom described a germline mutation in *PTEN* (Tyr68His) whom had right cortical macrocephaly with pachygyria and subcortical dysplasia into the V-SVZ (Jansen et al., 2015). These results are supported by a study that generated a knock-in mutation of threonine 366 to alanine in *PTEN* which demonstrated prevalent neuron cytomegaly, enhanced dendrite arborization, and behavioral changes (Ledderose et al., 2022). Thus, a single nucleotide change in *PTEN* has extensive neural effects. Therefore, detecting and delineating *de novo* somatic variants that are pathogenic, but account for a small variant allele frequency, may represent key challenges. One should also note that there are extensive challenges to detecting technical vs. biological variants and short read, long read, and single cell sequencing can all influence the capacity to detect mutations. Nevertheless, the prevalence of focal abnormalities does hint at plausible somatic mutation events. Subtle manifestations include focal dysplasia seen in patients with Cowden Syndrome or BRRS. An examples is that a case of a one-year-old that had HME subject to hemispherectomy had an indel and a 2-base duplication (Koboldt et al., 2021). Taken together, both PTEN and PI3K generation of lipids must be carefully controlled and mutations in these genes can lead to altered cortical development.

## Outlook

The overlapping clinical and anatomical phenotypes that have been long recognized in neurodevelopmental disorders has hinted at convergent pathogenic mechanisms. The fact that variants frequently influence the mTORC1 pathway components is perhaps not surprising given the highly evolutionarily conserved functions. Modeling somatic mosaicism has allowed for developing mechanistic insight into brain development and has yielded enthusiasm about potential therapies that target the mTORC1 pathway. In some cases, these therapies have been brought to clinic. Challenges that remain include developing non-invasive techniques to identify variant allele frequency in patients. In addition, long read technology with higher fidelity must be developed. In many patients, no mutations have been identified. It is unclear as to the frequency that abnormal cells cease to survive through apoptosis or clearance by immune cells such as microglia. The large number of non-pathogenic mutations may also help mold brain development in more subtle ways. Therefore, future experiments are needed to develop robust and high throughput examination of how a single variant in a gene, multiple changes in the same gene, or multiple changes among multiple genes may help to mold cerebrocortical cytoarchitecture and neural function.

## Figures and Tables

**TABLE 1:** Somatic Mosaicism.

Gene	Clinical Phenotype	Cellular Pathology	Mutation	Reference	Model	Model Pathology	Reference
*MTOR*	HME, HME with hypomelanosis of Ito, asymmetric megalencephaly with polymicrogyria and cutaneous pigmentary mosaicism	FCDII	c.4448C>T, p.Glu545Lys	Lee et al., 2012			
	HME, complex partial seizures	N.R.	p.C1483Y, p.A1669S	D’Gama et al., 2015			
	FCD	Cortical dyslamination and dysmorphic neurons, consistent with FCDIIa, Cortical dyslamination, dysmorphic neurons and balloon cells, consistent with FCDIIb	c.7280T>C p.Leu2427Pro, c.6577C>T p.Arg2193Cys, c.1871G>A p.Arg624His, c.5126G>A p.Arg1709His, c.6644C>T p.Ser2215Phe, c.7280T>A p.Leu2427Gln, c.5930C>A p.Thr1977Lys, c.4348T>G p.Tyr1450Asp, c.4447T>C p.Cys1483Arg, c.6644C>T p.Ser2215Phe, c.5930C>A p.Thr1977Lys	Lim et al., 2015	IUE Wild type or p.Leu2427Pro MTOR	Increased pS6, EN, CM, EEG seizures, behavioral seizures	Lim et al., 2015
	FCD, MEG-PMG, MEG	FCDIIa, increased pS6	c.4379T>C p.Leu1460Pro, c.6644C>T p.Ser2215Phe, c.6644C>A p.Ser2215Tyr, c.5930C>T p.Thr1977Ile, c.5395G>A p.Glu1799Lys	Mirzaa et al., 2016	rat neuron transfection	increased pS6 and CM	Mirzaa et al., 2016
	FCD, HME	FCDIIb	p.L1460P, p.S2215Y, p.S2215Y, p.S2215F, p.T1977R, p.T1977K, p.C1483R	D’Gama et al., 2017	EMX1-CRE, CamkII-CRE or IUE CRE Knockin inducible constituively active MTOR (quadruple mutant V2198A, L2216H, L2260P, I2017Y)	EMX1-CRE: HIF-1-associated apoptosis, Microcephaly, Degeneration IUE CRE: increased pS6, EN, CM CamkII-CRE: increased pS6, seizures, cytomegaly, macrocephaly	Kassai et al., 2014
	HME, FCD daily cluster-formed spasms with a suppression-burst pattern on electroencephalograph	FCD type IIa, characterized by many dysmorphic neurons with a unilayer pattern of the cortex and gliosis	c.4376C>A, p.Ala1459Asp	Hanai et al., 2017	IUE Mutant (MTOR A1459D) or WT MTOR	EN, CM	Hanai et al., 2017
	HME, FCD catastrophic epilepsy that is medically intractable, and surgical resection of the affected brain is necessary to alleviate epileptic episodes	cortical dyslamination with dysmorphic neurons and	C1483Y	Park et al., 2018	IUE Mutant (MTOR C1483Y)	EN, CM, EEG seizures, reduced cilia	Park et al., 2018
	HME with intractable epilepsy	CD, EN, CM, Gliosis	c.6644C > T:p.S2215F (somatic doublet with RPS6c.695G > A; p.R232H)	Pelorosso et al., 2019	IUE Mutant (RPS6 WT, RPS6 R232H, MTOR WT or MTOR S2215F)	Increased progenitor proliferation (mutant rpS6 and double mutant), EN (mutant MTOR and double mutant), CM (mutant MTOR and double mutant)	Pelorosso et al., 2019
	FCD, HME	FCDIIa, FCDIIb, HME with FCDIIb, Hemispheric FCDIIa	c.4376C>A/p.Ala1459Asp, c.4379 T>C/p.Leu1460Pro, c.5930C>A/p.Thr1977Lys, c.6644C>T/p.Ser2215Phe, c.6644C>A/p.Ser2215Tyr, c.7498A>T/p.Ile2500Phe	Baldassari et al., 2019b	IUE Cys1483Tyr, Leu2427Pro, or WT MTOR	Increased pS6, increased p4EBP, spontaneous seizures, altered mRNA translation	Kim et al., 2019
					IUE Mutant (Fat Domain L1460P and C1483Y) or kinase domain (I2017Y, V2198A, S2215Y, L2216H, L2260P, V2403F, E2419K, L2427T and L2431H) domains of mTOR, one triple mutant (V2198A, L2216H and L2260P) and one quadruple mutant (triple plus I2017Y), small deletion mutants missing two exons (del1418-90R) or one exon (del2434-56K)	Mutant dependent EN, CM	Tarkowski et al., 2019
*RHEB*	Synthetic mutation based upon evolutionary independence from *TSC1/2*	N/A	p. S16H	Yan et al., 2006	IUE Mutant (RHEB S16H)	increased pS6, increased p4E-BP, EN, CM, DH EEG seizures	Lin et al., 2016, Hsieh et al., 2016, Nguyen et al., 2019, Hsieh et al.,2020
	HME	FCDIIb	c.119A > T: p. Glu40Val	Salinas et al., 2019	IUE WT	EN, CM, DH	Moon et al., 2015, Reijnders et al., 2017, Sokolov et al., 2018
	ID syndrome associated with megalencephaly	N.R.	p.(Pro37Leu); (Ser68Pro) (Family of Doublet and one with Ser68Pro)	Reijnders et al., 2017	IUE Mutant (RHEB P37L)	EN, behvioral seizures	Reijnders et al., 2017
	HME with neonatal seizures (abnormal gyration characterized by the presence of multiple and small cortical gyri in the fronto-parietal lobes suggestive of polymicrogyria and a simplification and thickening of cortical gyri in the temporal lobe suggestive of lissencephaly. Furthermore, an abnormal signal over the white matter of fronto-parieto-occipital regions is suggestive of diffuse increased myelination, whereas poor gray white matter differentiation seems to be indicative of areas of cortical dysplasia. )	FCDIIb	c.119A> T: p.Glu40Val	Lee et al., 2021	IUE Mutant (RHEB P37L)	Increased pS6, EN, CM, DH, axon growth, EEG seizures	Onori et al., 2021
	FCD, HME	FCDIIb, HME with FCDIIb	c.119A>T/p.Glu40Val, c.[105C>A,104A > T]/p.Tyr35Leu	Baldassari et al., 2019b			
	intellectual delay with megalencephaly, FCD	FCDII	C105A/A104T; p.Y35L	Zhao et al., 2019	IUE Mutant (RHEB Y35L)	EN, CM, increased pS6, EEG seizures	Zhao et al., 2019
*TSC1*	TSC	(Cortical Tuber, subependymal nodule, SEGA, white matter nodules, migration tracks), glioais, hypomyelination	Inherited and Germline LOF	The European Chromosome 16 Tuberous Sclerosis Consortium	Conditional *Tscl* (Syn-Cre)	EN, CM, EEG seizures, MEG, increased pS6, hypomyleination	Kwiatkowski 2002, Meikle 2007, Wang 2007
	FCD	FCDIIb	p.Q55*	D’Gama et al., 2017	Conditional/Mutant *Tscl* (IUE CRE)	increased pS6, EN, CM, reduced latency to PTZ induced seizures	Feliciano et al., 2011
	FCD	FCDIIb	c.64C>T p.Arg22Trp; c.610C>T p.Arg204Cys	Lim et al., 2017	IUE CRISPR/Cas9 *Tscl*	EN, CM, EEG seizures	Lim et al., 2017
	FCD, Seizures	FCDIIb	c.1525C >T/p.Arg509, c.1907_1908delAG/p.Glu636fs*51	Baldassari et al., 2019b	Conditional *Tscl* (Nestin-rtTATetOp-cre)	EN, CM, increased pS6, giant cells, DH, EEG seizures, gliosis, MEG	Goto et al., 2011
	FCD	FCDIIb	c.64C > T; p.Arg22Trp, c.4258_4261delCAGT; p.Ser1420GlyfsTer55	Jha et al., 2022	Conditional *Tscl* (Emx1-CRE)	MEG, EEG seizures, CM, EN, increased pS6, gliosis, hypomyelination	Magri et al., 2011 and Carson et al., 2012
*TSC2*	TSC (Cortical Tuber, subependymal nodule, SEGA, etc)	(Cortical Tuber, subependymal nodule, SEGA, white matter nodules, migration tracks), glioais, hypomyelination	Inherited and Germline LOF	Van Slegtenhorst et al., 1997	Conditional *Tsc2* x hGFAP-CRE	EN, CM, EEG seizures, MEG, increased pS6, gliosis, hypomyleination	Hernandez et al., 2007, Gambello et al.,2009, Mietzsch et al., 2013
	FCD, HME infantile spasms or hypomotor, tonic, and clonic seizures	FCDIIb, HME	1 patient with germline missense variant p.L631P and a somatic missense variant p.E1558K. 1 patient with germline missense variant p.R1713H and a somatic frameshift variant p.Y587*, p.R751*	D’Gama et al., 2017	*Tsc2* (IUE shRNA)	Increased pS6, EN, CM	Tsai et al., 2014
	FCD (may have subpendymal heterotopia) in the right peri-trigone area, CM, EN, increased pS6	cortical dyslamination and dysmorphic neurons (consistent with FCDIIa), subependymal heterotopia	c.4639G>A p.Val1547Ile,	Lim et al., 2017	IUE CRISPR/Cas9 *Tsc2*	EN, CM, EEG seizures	Lim et al., 2017
	FCD	FCDIIa, FCDIIb	c.5228G >A/p.Arg1743Gln, 2380C>T/p.Gln794*, c.3725dupA/p.Glu1243fs	Baldassari et al., 2019b	*Tsc2*−/− embryos	exencephaly, thinning of the neuroepithelium, embryonic lethality	Onda et al., 2002
	FCD	FCDIIa, FCDIIb	c.64C > T; p.Arg22Trp, c.4258_4261delCAGT; p.Ser1420GlyfsTer55	Jha et al., 2022			
*DEPDC5*	FCD	FCDIIa	p.R874* (Germline)	D’Gama et al., 2017	Depdc5−/− and +/− rat c.40_44delins17/p.Gly15*or c.39_55delinsT/p.Lys13fs*8	Depdc5−/− embryonic lethality starting at E14.5, growth delay, reduced V-SVZ thickness, LGE size, and brain ventricle size. Increased neuron pS6 and size. Hets do not have seizures.	Marsan et al., 2016
	familial focal epilepsy with variable foci	N.R.	c.1122delA p.Leu374Phefs*30, c.715C>T p.Arg239*, c.982C>T p.Arg328*, c.1114C>T .Gln372*, c.1454G>A p.Arg485Gln, c.4567C>T p.Gln1523*	Ishida et al., 2013	Depdc5+/− and −/− mice	Heterozygous mice appear normal. Homozygotes display severe phenotypic defects between 12.5-15.5 dpc, including hypotrophy, anaemia, oedema, and cranial dysmorphology as well as blood and lymphatic vascular defects. Abnormal cortical development.	Hughes et al., 2017
	sleep-related frontal lobe epilepsy and FCD	FCDIIa with increased neuron pS6	Inherited c.856C>T/p.Arg286* with c.865C>T/p.Gln289* with somatic second hit	Ribierre et al., 2018	IUE CRISPR/Cas9	EN, CM, increased pS6, DH, increased spine width, EEG seizures	Ribierre et al., 2018
	Autosomal dominant familial focal epilepsy with variable foci	N.R.	c.4397G>A p.Trp1466*, c.1459C>T p.Arg487*, c.2527C>T p.Arg843*, c.4397G>A p.Trp1466*, c. 3802C>T p.Arg1268*, c.3311C>T p.Ser1104Leu, c.3217A>C p.Ser1073Arg, c.1355C>T p.Ala452Val, c.193 +1G>A, c.279+1G>A	Dibbens et al., 2013	IUE CRISPR/Cas9 and talen Depdc5−/− vs. +/− mouse	EN, CM, increased pS6, EEG seizures	Hu et al., 2018
	FCD	FCDIIa, FCDIIa (hemispheric)	DEPDC5: c.3021 + 1G >A (Germline+LOH), Several Germline	Baldassari et al., 2019b	DEPDC5f/f (Syn-CRE)	increased pS6, MEG, CM, gliosis, 60 days develop hunched back and evidence of neurologic dysfunction by limb-clasping behavior (hind limb strain), median survival of 105 days, behavioral seizures (not recorded by EEG), reduced latency to PTZ induced seizures	Yuskaitis et al., 2018
	HME	HMEG, FCDIIa	c.4187delC, p.Alal396Valfs*7 8	Mirzaa et al., 2016	Depdc5f/mutant (IUE CRE) (Mutant allele from Hughes 2017)	increased pS6, EN, CM, DH, behavioral seizures	Dawson et al., 2020
	FCD	FCDI, FCDIIa	c.715C>T (p.Arg239*) and c,1264C>T (p.Arg422*) (Two Hit), c.484-lG>A, c,1264C>T (p.Arg422*), c,1759C>T (p.Arg587*)	Baulac et al., 2015	Depdc5+/− vs RNAi mouse neurons	Increased pS6, CM, DH for RNAi. No change in pS6 for Depdc5+/− or cell size but DH.	De Fusco et al., 2020
	lesional and nonlesional epilepsies with focal dysplasias (bottom of the sulcus) or focal band heterotopia	N.R.	c.418C>T; p.Gin 140*, Germline	Scheffer et al., 2014	DEPDC5f/f (EMX1-CRE)	increased pS6, CM, MEG, premature mortality, EEG seizures	Ishida et al., 2022
*NPRL2*	FCD	left superior frontal gyrus FCD Ha	p.Q188* (Germline)	D’Gama et al., 2017	Nprl2f/f (EMX1-CRE)	increased pS6, CM, MEG, premature mortality, EEG seizures	Ishida et al., 2022
	Epilepsy	N.R.	c.100C>T p.Arg34* c.1134C>G p.Cys378Trp c.683+lG>C p.(?)	Baldassari et al., 2019b			
*NPRL3*	focal epilepsy, FCD	FCDIIa, increased pS6	III-4: c. 1375_1376dupAC, p.S460Pfs*20,c.l352-4delACAGinsTGACCCATCC, c.275G>A, p.R92Q	Sim et al., 2016	Nprl3f/f (EMX1-CRE)	increased pS6, CM, MEG, premature mortality, EEG seizures	Ishida et al., 2022
	Epilepsy	N.R.	c.301C>T p.GlnlOl* c.493delC p.Argl65Glyfs*5 c.562C>T p.Glnl88* c,1270C>T p.Arg424* c,1557C>G p.Tyr519* Deletion (exons 5-10) p.(?) Deletion (exons 1-7) p.(?)	Baldassari et al., 2019b			
	heterogenous seizures (focal, generalized, infantile spasms, febrile)	N.R.	c.349delG, p.Glull7LysFS;	Iffland et al.,2022	CRISP R/Cas9	increased pS6, CM, EN, EEG seizure	Iffland et al.,2022
*AKT3*	HME	neuronal heterotopia	49C>T;Glul 7Lys	Lee et al., 2012			
	HME, FCD, bilateral cortical malformation, polymicrogyria, periventricular nodular heterotopia, diffuse megalencephaly	aberrant surface contour with thick cortical ribbon and molecular layer, numerous subcortical bands and islands of ectopic gray matter that contain neurons and glia, collections of neuroblast-like cells (microdysplasia), ectopically dividing cells with atypical nuclei within gray and white matter	trisomy 1q, c.49G>A p.E17K	Poduri et al., 2012			
	Overlap of Phenotypes, MPPH	N.R.	c.686A>G, p.Asn229Ser, c.1393C>T p.Arg465Trp (Germline)	Riviere et al., 2012			
	HME	FCD type IIa with cortical dyslamination, blurring of the grey-white junction, and dysmorphic neurons. No balloon cells	p.Glu17Lys	Jansen et al., 2015			
	HME	FCD dysorganization across the entire hemisphere	c.49G>A; p.E17K	Baek et al., 2015	IUE Mutant (AKT3 E17K)	EN, CM, increased pS6, EEG seizures	Baek et al., 2015
	FCD, HME	FCDIIa, HME/FCDII	c.49G>A/p.Glu17Lys	Baldassari et al., 2019b			
	HME	N.R.	p.E17K	D’Gama et al., 2017			
	DME/HME	N.R. for mosaic. Germline p.R465W 6 year old with 2x brain weight of adult brain, asymmetrically enlarged with diffuse cortical dysplasia with irregular hyperconvoluted gyri, anomalous branching and fusion of gliotic layer 1, more layer 6 and white matter neurons which appeared disorganized and maloriented. Neurons did not appear enlarged or dysplastic, and no balloon cells were identified.	c.49G>A p.Glu17Lys, Several Germline	Alcantara et al., 2017			
*AKTl*	Proteus syndrome with HME	N.R.	p.E17K	D’Gama et al., 2017			
*PIK3R2*	Megalencephaly-Polymicrogyria-Polydactyly-Hydrocephalus Syndrome	N.R.	c.1117G>Ap.Gly373Arg (Germline)	Riviere et al., 2012	PIK3R2 p.G367R Mutant Mice	MEG, mild EN, CM, increased pS6, EEG seizures	Shi et al., 2020
	Bilateral perisylvian polymicrogyria	N.R.	p.Gly373Arg	Mirzaa et al., 2015			
	PMG, macrocephaly	N.R.	p.K376E (Germline)	D’Gama et al., 2017			
*PIK3R3*	FCD	FCDI	N.R.	Chung et al., 2023			
PIK3CA	HME	CD, CN, EN, PMG	1633G>A; Glu545Lys	Lee et al., 2012			
	Megalencephaly-capillary malformation	N.R.	p.Glu81Lys, p.Arg88Gln, p.Cys378Tyr, p.Glu726Lys, p.Gly914Arg, p.Thr1025Ala (All Germline)	Riviere et al., 2012	EMX1-CRE or NKX2.1-CRE x conditional PIK3CAH1047R Knockin	MEG, abnormal gyrification in cortex, abnormal lamination especially in superficial layers	D’Gama et al., 2017
	HME, DME, FCD	FCDIIa	p.His1047Arg, p.Thr544Asn, p.His1047Arg	Jansen et al., 2015			
	DME, HME, FCD	FCDIIa	c.1624G>A/p.Glu542Lys, c.3140A>G/p.His1047Arg	Baldassari et al., 2019b			
	HME	N.R.	p.E545K	D’Gama et al., 2015			
	HME	N.R.	p.E542K	D’Gama et al., 2017			
*PTEN*	Tumors (Also Bannayan-Riley-Ruvalcaba syndrome, Cowden syndrome, multiple hamartoma syndrome, and proteus-like syndrome)	N.A.	Loss of heterozygosity (LOH) at chromosome 10q23	Li et al., 1997	Ptenf/f (GFAP-CRE)	CM, ENs, MEG and behavioral seizures	Backman et al., 2001; Kwon et al., 2001
	HME with pachygyria and subcortical dysplasia		p.Tyr68His (Germline)	Jansen et al., 2015	Ptenf/f (NSE-CRE)	CM, ENs, MEG, DH, increased pS6, premature mortality, EEG seizures,	Kwon et al., 2006
					IUE CRISPR/Cas9	ENs, CM	Chen et al., 2015
	HME	Anterior cerebrum with complex sulcal pattern including small gyri and occasional gyral fusion across sulci. Ectopic neurons, mislamination,the posterior cerebrum had a flat, simplified cortical surface with thick cortical ribbon and neurons were dysmorphic with cytomegally and irregular processes. Reduced neuronal heterogeneity with dysmorphic and non-dysmorphic neurons that were malpositioned.	c.255_262delTGCACAATinsC; c.1110_1111dupTG	Koboldt et al., 2021	Inducible PTEN T366A (AAV1-CRE in neonates)	Increased pS6, CM, DH	Ledderose et al., 2022
	Right perisylvian dysplasia with globally reduced myelination and periventricular gliosis on the right side. Dysplastic and harmartomatous appearance to the cerebellum		p.385G>A, p.G129R	Elia et al., 2012			

The table includes key studies and their identification of gene mutations and the resultant abnormal cerebro-cortical development on the left-hand side. On the right-hand side are seminal studies that generated animal models of malformations of cortical development to study identified mutations. Both the genetic studies and animal models list specific phenotypes when indicated. Mutations are denoted by c. which indicates DNA base followed by original base number and DNA base mutated to. p. denotes protein followed by original amino acid, position, and amino acid or protein change. Del, deletion *, nonsense Clinical Phenotype: HME, Hemimegalencephaly, FCD, Focal Cortical Dysplasia, MEG-PMG, Megalencephaly-polymicrogyria, ID, Intellectual Disability, DME, Diffuse Megalencephaly, MEG,Megalencephaly, pS6,phospho-ribosomal S6 subunity, EEG,Electroencephalogram, EN, Ectopic Neuron, CM, Cytomegaly, DH, Dendrite Hypertrophy, WT, Wild-type, N.R., Not Reported. Not all studies listed DNA base mutation, clinical phenotype, or cellular pathology. Note that some studies identified inherited or *de novo* constitutional mutations while others identify somatic mutations. Seizures were reported for most studies with malformations present. Seizures were noted in several cases listed in the absence of clinical cortical malformations.
